# TSG-6 in conditioned media from adipose mesenchymal stem cells protects against visual deficits in mild traumatic brain injury model through neurovascular modulation

**DOI:** 10.1186/s13287-019-1436-1

**Published:** 2019-11-05

**Authors:** Kumar Abhiram Jha, Mickey Pentecost, Raji Lenin, Jordy Gentry, Lada Klaic, Nobel Del Mar, Anton Reiner, Chuan He Yang, Lawrence M. Pfeffer, Nicolas Sohl, Rajashekhar Gangaraju

**Affiliations:** 1 Department of Ophthalmology, University of Tennessee Health Science Center, College of Medicine, 930 Madison Ave, Suite#768, Memphis, TN 38163 USA; 2 Cell Care Therapeutics, Inc., Los Angeles, CA USA; 3 Present Address: Pathways to Stem Cell Science, Monrovia, CA USA; 4 Department of Anatomy and Neurobiology, University of Tennessee Health Science Center, College of Medicine, 855 Monroe Avenue, Suite#515, Memphis, TN 38163 USA; 5 Department of Pathology, University of Tennessee Health Science Center, College of Medicine, 19 South Manassas Street, Suite#214, Memphis, TN 38163 USA

**Keywords:** Endothelial, ERG, OKN, Retina, Microglia, Muller, Paracrine, TBI, MSC, SOD2, Stat3

## Abstract

**Background:**

Retinal inflammation affecting the neurovascular unit may play a role in the development of visual deficits following mild traumatic brain injury (mTBI). We have shown that concentrated conditioned media from adipose tissue-derived mesenchymal stem cells (ASC-CCM) can limit retinal damage from blast injury and improve visual function. In this study, we addressed the hypothesis that TNFα-stimulated gene-6 (TSG-6), an anti-inflammatory protein released by mesenchymal cells, mediates the observed therapeutic potential of ASCs via neurovascular modulation.

**Methods:**

About 12-week-old C57Bl/6 mice were subjected to 50-psi air pulse on the left side of the head overlying the forebrain resulting in an mTBI. Age-matched sham blast mice served as control. About 1 μl of ASC-CCM (siControl-ASC-CCM) or TSG-6 knockdown ASC-CCM (siTSG-6-ASC-CCM) was delivered intravitreally into both eyes. One month following injection, the ocular function was assessed followed by molecular and immunohistological analysis. In vitro, mouse microglial cells were used to evaluate the anti-inflammatory effect of ASC-CCM. Efficacy of ASC-CCM in normalizing retinal vascular permeability was assessed using trans-endothelial resistance (TER) and VE-cadherin expression in the presence of TNFα (1 ng/ml).

**Results:**

We show that intravitreal injection of ASC-CCM (siControl-ASC-CCM) but not the TSG-6 knockdown ASC-CCM (siTSG-6-ASC-CCM) mitigates the loss of visual acuity and contrast sensitivity, retinal expression of genes associated with microglial and endothelial activation, and retinal GFAP immunoreactivity at 4 weeks after blast injury. In vitro, siControl-ASC-CCM but not the siTSG-6-ASC-CCM not only suppressed microglial activation and STAT3 phosphorylation but also protected against TNFα-induced endothelial permeability as measured by transendothelial electrical resistance and decreased STAT3 phosphorylation.

**Conclusions:**

Our findings suggest that ASCs respond to an inflammatory milieu by secreting higher levels of TSG-6 that mediates the resolution of the inflammatory cascade on multiple cell types and correlates with the therapeutic potency of the ASC-CCM. These results expand our understanding of innate mesenchymal cell function and confirm the importance of considering methods to increase the production of key analytes such as TSG-6 if mesenchymal stem cell secretome-derived biologics are to be developed as a treatment solution against the traumatic effects of blast injuries and other neurovascular inflammatory conditions of the retina.

## Introduction

According to the Defense and Veterans Brain Injury Center (DVBIC) of the Department of Defense, a staggering 383,947 US military service members sustained TBI in 2018 of which 82.3% of them were classified as mild TBI (mTBI) [1]. While the majority of recorded mTBI cases are related to blast injuries during active duty, some occurred while playing sports or other recreational activities. Despite the implementation of awareness programs and efforts to improve protective equipment to protect those at high risk of blast injuries, the face and brain remain highly vulnerable [2]. Furthermore, secondary displacement of the brain during injury can progress into exacerbated neuropathology and visual deficits that often correlate to acceleration-deceleration concussive insults to optic nerve [3]. Inflammation, oxidative stress, and subsequent cell signaling disturbances stemming from the acceleration-deceleration are causally linked to such neuropathology [4, 5]. We and others have shown that activation and proliferation of microglial cells are associated with a cycle of inflammation and leads to vascular damage, cell death, and subsequent loss of retinal barrier integrity [6–11]. Consequently, therapies aimed at controlling microglial activation are urgently needed.

Using a validated preclinical animal model of mTBI, we previously reported the beneficial effects of using the secretome of adult mesenchymal stem cells (MSCs) derived from the stromal vascular fraction of human adipose tissue (adipose stem/stromal cells (ASC)) [11]. The concentrated conditioned media from adipose tissue-derived mesenchymal stem cells (ASC-CCM) that have been primed with TNFα and IFNγ release a variety of cytokines and chemokines among which are extracellular superoxide dismutases (SOD2 and SOD3), immune modulatory proteins like indoleamine 2,3-dioxygenase (IDO), and TNF-stimulated gene 6 protein (TSG-6). Individually, these proteins have been shown to have therapeutic value in numerous animal models, including ophthalmic disease models [11–15]. Our studies indicate that upregulated proteins in the ASC-CCM when ASCs are pre-stimulated with inflammatory cytokines can suppress microglial activation and protect retinal barrier integrity. However, among the many proteins released by ASCs, there remains a need to better understand the role of specific proteins responsible for the observed in vivo therapeutic effect. TSG-6, a multifunctional glycoprotein, has emerged as a potential regulator of inflammation in a variety of diseases including diabetes, corneal injury, asthma, acute pancreatitis, and brain injury [16–20]. Observations that bone marrow MSC-derived TSG-6 inhibit the LPS-mediated pro-inflammatory activation of BV2 cells, a murine microglia-like cell line, point to an important protective role for TSG-6 and led us to focus on understanding the function and effect of mesenchymal stem cell-secreted TSG-6 [21]. Our experiments illustrate that TSG-6 impacts microglia in our mTBI model.

In this study, we generated concentrated conditioned media from ASCs that were treated with siRNA against TSG-6 or a non-targeting control and stimulated with cytokines to determine whether TSG-6 is responsible for the rescue in the visual deficits in our mTBI mouse model [4, 22]. Furthermore, we have evaluated the effects of TSG-6 on BV2 cells and retinal endothelial cells in vitro. Our data demonstrate TSG-6-dependent suppression of microglial activation in vivo and protection against retinal barrier integrity in vitro suggesting a key role for TSG-6 in ASC-CCM.

## Materials and methods

### TSG-6 knockdown in ASC culture and conditioned medium preparation

Commercial ASCs were purchased from Lonza (Walkersville, MD, USA; Cat# PT-5006, Lot# 0000535975). To knock down TSG-6 expression, ASCs were seeded into T75 flasks at a density of 15,000 cells/cm^2^ at 37 °C and 5% CO_2_ in 15 ml of complete media (MEM-alpha with 10%FBS). At 8 h post-seeding, cells were transfected for 16 h with negative control siRNA (Cat#: 4390844, Ambion) or a validated siRNA against TSG-6 (Cat#4392420, Ambion) using Lipofectamine® RNAiMAX reagent (Invitrogen, Cat#: 13778150). ASCs were subsequently washed twice with DPBS and then primed with 20 ng/ml TNFα (R&D Systems) and 10 ng/ml IFNγ (R&D Systems) in basal media for 24 h. ASCs were again washed twice with DPBS to remove cytokines and then cultured in basal media for an additional 24 h at which point that the conditioned media were collected and the cells were lysed in 1.5 ml of 100 mM Tris-HCL pH 8.0, 150 mM NaCL, 5 mM EDTA, 5% glycerol, and 0.1% NP40 supplemented with Halt™ Protease Inhibitor Cocktail (Thermo Fisher Scientific). The conditioned media were filtered through a 0.45-μm syringe filter and then concentrated ~ 20× using a 3-kDa molecular weight cutoff Amicon Ultra-15 centrifugal concentrator and desalted by the addition and concentration of 14 ml DPBS twice. The total amount of protein in the desalted, concentrated conditioned medium samples (primed or unprimed siControl-ASC-CCM and siTSG-6-ASC-CCM) were measured using the Qubit Protein Assay Kit and a Qubit fluorimeter. Aliquots were stored at − 80 °C and normalized to the same concentration with DPBS for all comparative assays.

### Western blot analysis

Concentrated conditioned medium samples and cell lysates were assessed by immunoblot analysis for TSG-6, COX IV, and TIMP-1 as described by us previously [11]. Total STAT3 and phosphorylated STAT3 (pY705-STAT3) were assessed as we previously described [23].

### Nitric oxide release assay

The nitrite concentration in microglial cell culture media pre-incubated with primed siControl ASC-CCM or primed siTSG-6 ASC-CCM was determined using the Greiss Reagent System as described previously [11].

### Microglial and retinal endothelial cell culture and activation

The mouse microglial cell line, BV2, was a kind gift from Professor Grace Sun, Ph.D., University of Missouri, Columbia, MO. Activation of BV2 cells was performed as we described previously [11]. Human retinal endothelial cells (HREC; Cell Systems, Inc.) were seeded at a density of 4 × 10^4^ cells/cm^2^ and grown for 24 h in growth media. Cells were switched to serum-free media, preincubated with and without primed siControl-ASC-CCM or primed siTSG6-ASC-CCM (100 μg/well) for 6 h, and then activated with TNFα (1 ng/ml; Sino Biological, Inc.) for 18 h and proceeded with Western blot analysis.

### Gene expression analysis

Retinal extracts from TBI mice were assessed for gene expression changes using NanoString Counter® multiplex analysis with the neuropathology panel as we previously described [23]. Briefly, in each group, four to nine individual mouse retinas were analyzed 4 weeks post-blast injury. The nCounter Dx Analysis System uses gene-specific probe pairs that hybridize directly to mRNA samples. A reporter probe carries the fluorescent signal; a capture probe allows the complex to be immobilized for data collection. Analysis of data QC, normalization, and differential gene expression data was analyzed using nSolver 4.0 with nCounter Advanced Analysis (Version 2.1.115). The expression levels of representative gene transcripts (Table 1) were further confirmed by real-time quantitative PCR using TaqMan assays and analyzed using 2−DDCt method. Gene expressional analysis carried out using total RNA isolated from BV2 cells was described by us recently [11].

**Table 1:** Expression levels of representative gene transcripts

Genes	TaqMan assay ID	Reference sequence
18S ribosomal RNA (18s)	Mm04277571_s1	NR_003278.3
Interleukin 1 beta (Il1β)	Mm00434228_m1	NM_008361.3
Cluster of differentiation 86 (Cd86)	Mm00444543_m1	NM_019388.3
Glial fibric acid protein (GFAP)	Mm01253030_m1	NM_001131020.1
Interferon regulatory factor 8 (Irf8)	Mm00492567_m1	AK018533.1
v-erb-b2 erythroblastic leukemia viral oncogene homolog 3 (Erbb3)	Mm01159999_m1	NM_010153.1
Fas/TNF receptor superfamily member 6 (Fas)	Mm01204974_m1	NM_007987.2
Vascular cell adhesion molecule 1 (Vcam1)	Mm01320973_m1	NM_011693.3
Endothelin 2 (Edn2)	Mm00432983_m1	NM_007902.2

### Immunocytochemistry

Immunocytochemistry was performed to reveal the localization of Iba1 and F-actin in the BV2 cells as described by us recently [11]. Quantification of pixel intensities (relative fluorescence units) for Iba1 was analyzed from at least five images from different locations per well and expressed as an average of the experimental groups. Immunocytochemistry for VE-cadherin in HREC was performed by a method as we described previously [24].

### Retinal endothelial cell permeability in vitro

Measurements of trans-endothelial electrical resistance (TER) were performed according to our previously published method that utilizes the measurement of electric cell-substrate impedance sensing (ECIS; Applied Biophysics, Troy, NY) [11]. Changes in the resistance upon exposure to TNFα (1 ng/ml) were monitored for up to 18 h with siControl-ASC-CCM or siTSG-6-ASC-CCM. Resistance values at 4000 Hz for multiple wells were normalized to an identical starting resistance value, averaged, and presented as normalized resistance over time.

### Animals and blast injury study groups

The TBI mouse model was performed as described previously [4]. Experiments were performed in two batches; eight animals in the sham blast group received saline; eight animals in the 50-psi blast group received saline; ten animals in the 50-psi blast group received 1 μl (~ 68 μg/ml of total protein) of primed siControl-ASC-CCM; and ten animals in the 50-psi blast group received 1 μl (~ 68 μg/ml of total protein) of primed siTSG-6-ASC-CCM. Intravitreal injections were performed with a 30-gage microsyringe (Hamilton, Reno, NV), on the temporal side of the eye, 2 mm posterior and parallel to the limbus. Various analyses were performed 4 weeks after the intravitreal injections (Additional file 1: Figure S1).

### Optokinetic reflex measurements

To assess visual function, optokinetic reflex measurements were made 4 weeks post-blast injury as we described previously [11]. Visual acuity was assessed at 100% contrast by varying spatial frequency threshold. Contrast sensitivity was assessed in mice by varying the contrast at 0.042 cycles per degree (c/d) of the spatial frequency threshold.

### Electroretinogram measurement

Scotopic threshold electroretinogram (ERG) recordings were obtained using the Espion E2 ERG system (Diagnosys LLC, Lowell, MA) as we described previously [25]. Mice were presented with different flashes of increasing intensity (0.0025, 0.025, 0.25, 2.5, 25 cd s m^2^), each repeated five times, with an inter-stimulus interval ranging from 20 s for dim flashes to 1 min for the brightest flashes. Three to five ERG traces at each flash luminance were averaged to measure b-wave amplitude.

### Tissue preparation and immunohistochemistry

Post-euthanasia eyes from all groups were enucleated, and retinal eyecups were processed for immunohistochemical analysis of GFAP expression as we described previously [11]. Quantification of pixel intensities (relative fluorescence units) of GFAP-immunolabeled Müller cells was analyzed from at least three sections (from NFL to RPE) per eye, three areas per retina (two mid-peripheral and a central) by an investigator blinded to the groups and expressed as mean intensity per 100,000 μm^2^ of the retina.

### Statistical analysis

Results are expressed as mean ± SEM for all experiments. Pairwise *t* tests were run in order to calculate the *p* values for comparisons between the individual groups. One way ANOVA followed by post hoc *t* tests with the Bonferroni correction was used for multiple group comparisons using GraphPad Prism software. For NanoString analysis, the signal intensities (arbitrary units) for each target from four to nine individual retina were averaged, and pairwise group comparisons were made. Any expression below 0.5-fold was considered downregulated; 0.5–1.5-fold considered unchanged, and any value above 1.5-fold was considered upregulated. All samples with correlation coefficients > 0.8 within the biological group were included in the study. Heat maps were generated by unsupervised clustering analyses using Spearman correlation in the nSolver program. In all analyses, a *p* value < 0.05 was considered statistically significant.

## Results

### Depletion of TSG-6 from the concentrated conditioned medium from cytokine primed ASCs

**Fig. 1:**
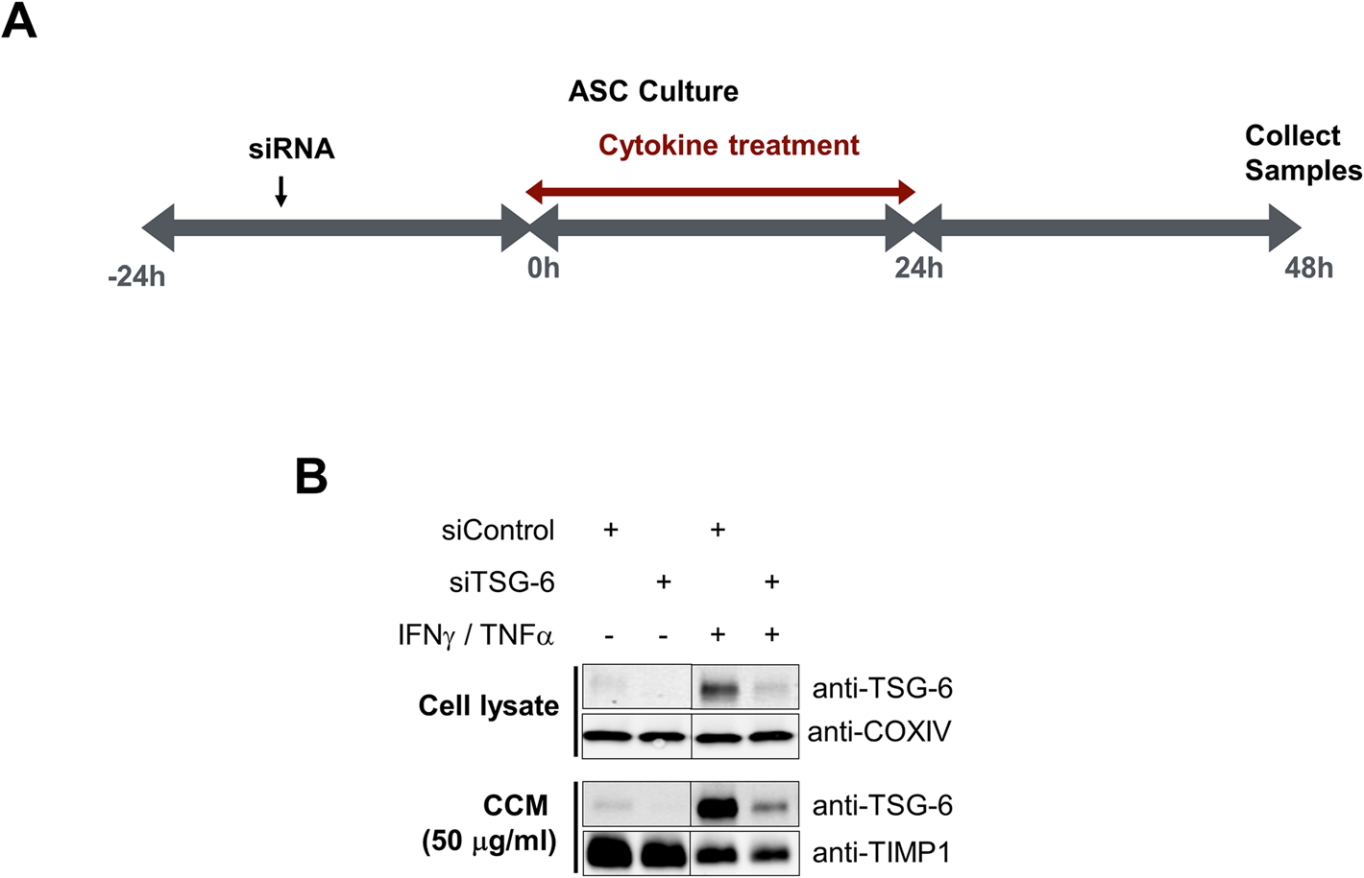
Depletion of TSG-6 from cytokine-primed ASC conditioned medium. **a** Schema for preparation of siRNA-mediated knockdown of TSG-6 in conditioned medium from exogenous cytokine-stimulated ASCs. **b** Immunoblot analysis of TSG-6 in cell lysates and CCM. COXIV and TIMP1 in CCM remained unchanged. Data represent mean ± SEM from at least three technical replicates

Previously, we have shown that TSG-6 secretion by ASCs primed with inflammatory cytokines continued after their removal, allowing for the collection of anti-inflammatory conditioned media [11]. In this study, ASCs were first treated with TSG-6 siRNA or control siRNA and then primed with IFNγ and TNFα in serum-free media to permit conditioning of serum- and cytokine-free media with the cell secretome according to the schema in Fig. 1a and as described in the “Materials and methods” section. The conditioned media were collected, filtered to remove cell debris, and then concentrated and desalted using 3-kDa molecular weight cutoff centrifugal filters. Concurrently, the cells were lysed for Western blot analyses. As shown in Fig. 1b, transient transfection of ASCs with TSG-6 siRNA resulted in a significant reduction in TSG-6 levels both in the cell lysates and concentrated conditioned media as compared to cells that were treated with control siRNA and primed with cytokines. COXIV served as an internal control for cell lysates. TIMP1 served as an internal control for secreted proteins in the concentrated conditioned media. Both analytes were unaffected with TSG-6 knockdown suggesting no adverse effect of TSG-6 knockdown in ASCs. Moreover, the specificity of TSG-6 knockdown was evidenced by the levels of IDO1 and SOD2 being upregulated by cytokine treatments but unaffected by siRNA treatments (Additional file 2: Figure S2).

### TSG-6-depleted ASC-CCM fails to suppress microglial activation

**Fig. 2:**
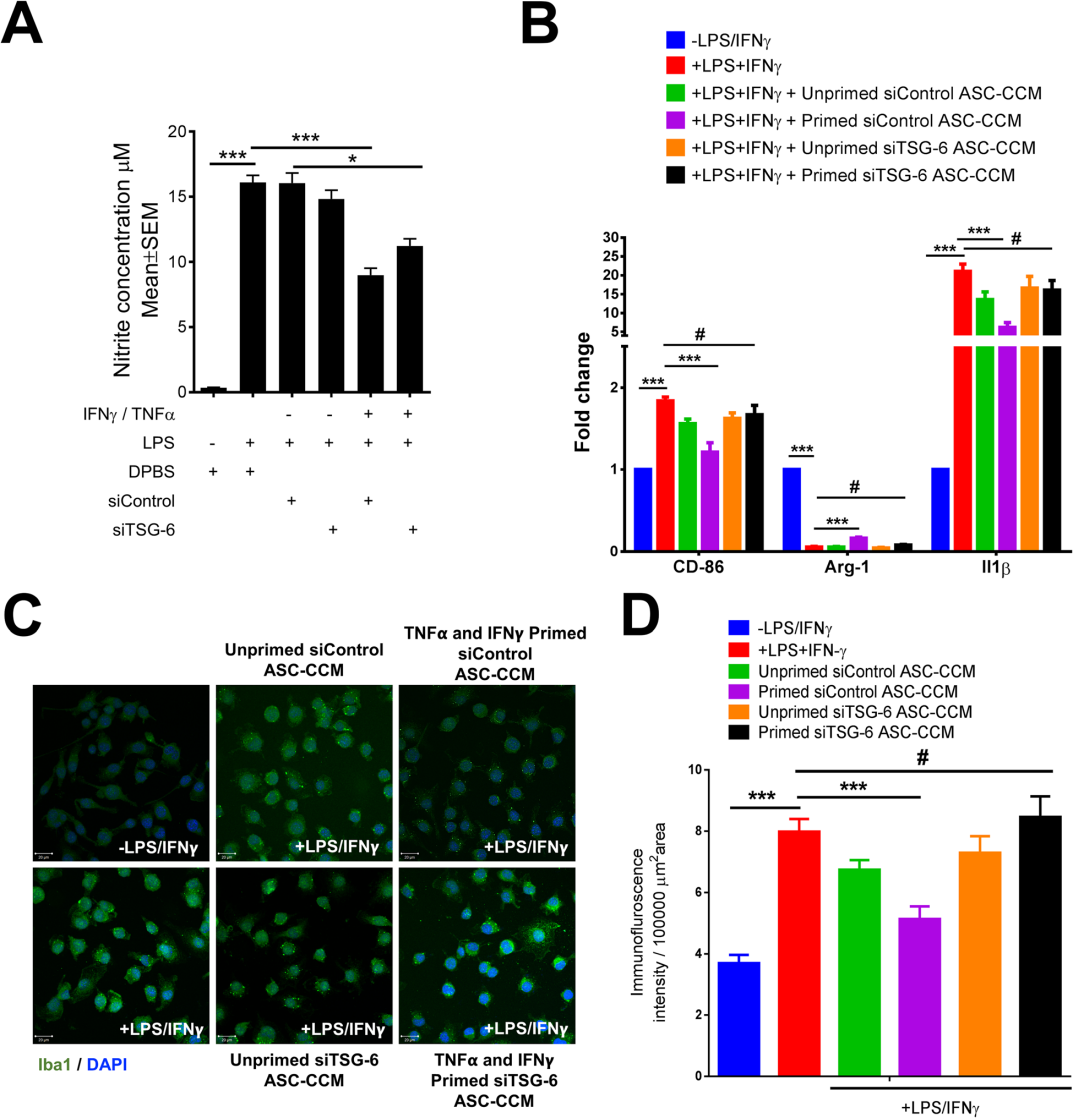
Depletion of TSG-6 from ASC-CCM fails to suppress microglial activation. **a** Biochemical assessment of NO release from BV2 cells using the Griess assay. **b** Assessment of gene expression by Sybr Green real-time PCR and expressed as fold change normalized to internal control (GAPDH) in the study groups. Data represent mean ± SEM from three separate experiments performed in duplicate. *p < 0.05; ***p < 0.001; ^#^*p* > 0.05. **c** Microglial activity as shown by the increased Iba1 immunoreactivity with LPS and IFNγ-stimulated BV2 cells after 12 h exposure. The bar graph shows the quantification of mean fluorescence intensity of Iba1. Data are mean ± SEM performed in duplicates. ^#^*p* > 0.05; ***p < 0.001. Scale bar = 20 μm

We previously showed that ASC-CCM could inhibit the LPS-mediated pro-inflammatory activation of BV2 cells, a murine microglia-like cell line [11]. To address the role of TSG-6 in these observed effects, we performed experiments with BV2 cells. While primed siControl-ASC-CCM could suppress the production of nitrite by LPS-treated BV2 cells, primed siTSG-6-ASC-CCM at the same total protein concentration (5 μg/ml) failed to suppress nitrite release (*p* < 0.01, Fig. 2a). Interestingly neither unprimed siControl-ASC-CCM nor unprimed siTSG-6-ASC-CCM could suppress nitrite release, confirming our previous observations that cytokine priming is essential to generating sufficient release of TSG-6 by ASCs for therapeutic effect.

Since the production and release of cytokines play a central role in the microglia-mediated inflammatory action and their regulation of gene expression, we next assessed the expression of IL-1β and CD-86 (early and late markers of the M1 phenotype of microglia) and arginase-1 (a marker of M2 phenotype of microglia) by real-time PCR. Whereas the gene transcripts of IL-1β (*p* < 0.001) and CD-86 (*p* < 0.001) was significantly increased in BV2 cells treated with LPS and IFNγ, the expression of Arg-1 decreased (*p* < 0.001) compared to untreated cells confirming the activation of BV2 cells. In agreement with the nitrite release data, only primed siControl-ASC-CCM significantly reduced IL-1β (*p* < 0.001), CD-86 (*p* < 0.001), and Arg-1 (*p* < 0.001) gene expression (Fig. 2b), while primed siTSG-6-ASC-CCM failed to suppress gene expression in BV2 cells. Again, neither unprimed siControl-ASC-CCM nor unprimed siTSG-6-ASC-CCM could suppress BV2 gene expression.

### TSG-6-depleted ASC-CCM fails to preserve resting cell morphology in LPS and IFN-γ-stimulated BV2 cells

Previously, we have shown that ASC-CCM could preserve the resting cell morphology of BV2 cells subsequent to microglial activation [11, 26]. To address the role of TSG-6 in this pathway, we assessed the morphological changes in BV2 cells by inverted phase contrast microscope and F-actin immunostaining. While primed siControl-ASC-CCM preserved the morphology of BV2 cells with well-defined soma with distal arborization similar to control cells, cells treated with primed siTSG-6-ASC-CCM failed to preserve the morphology and were morphologically similar to BV2 cells treated with LPS and IFNγ with fewer branches, predominately appearing amoeboid (Additional file 3: Figure S3). Neither unprimed siControl-ASC-CCM nor unprimed siTSG-6-ASC-CCM affected the morphology of activated BV2 cells. Previously, we have also shown a correlation of BV2 cell activation with increased ionized calcium-binding adapter molecule 1 (Iba1) immunostaining [11, 27]; here, we assessed the role of TSG-6 in the increased Iba1 staining. BV2 cells treated with LPS and IFNγ demonstrated an increase in Iba1 immunostaining. In contrast, cells that were pre-incubated with primed-siControl-ASC-CCM, but not primed-siTSG-6-ASC-CCM, demonstrated a decrease in Iba1 immunoreactivity (Fig. 2c). The mean total pixel intensity of Iba1 expression in the control group was 3.7 ± 0.26, while the pixel intensity of Iba1 expression in cells treated with LPS/ IFNγ was 7.98 ± 0.41 (mean intensity/10,000 μm^2^; *p* < 0.001). Cells pretreated with primed siControl-ASC-CCM and then treated with LPS/ IFNγ showed reduced Iba1 expression (5.14 ± 0.4 mean intensity/10,000 μm^2^; *p* < 0.001) but not cells pretreated with primed siTSG-6-ASC-CCM (8.46 ± 0.67 mean intensity/10,000 μm^2^; *p* < 0.05; Fig. 2d).

### TSG-6-depleted ASC-CCM fails to protect against TNFα-induced loss of endothelial barrier integrity

**Fig. 3:**
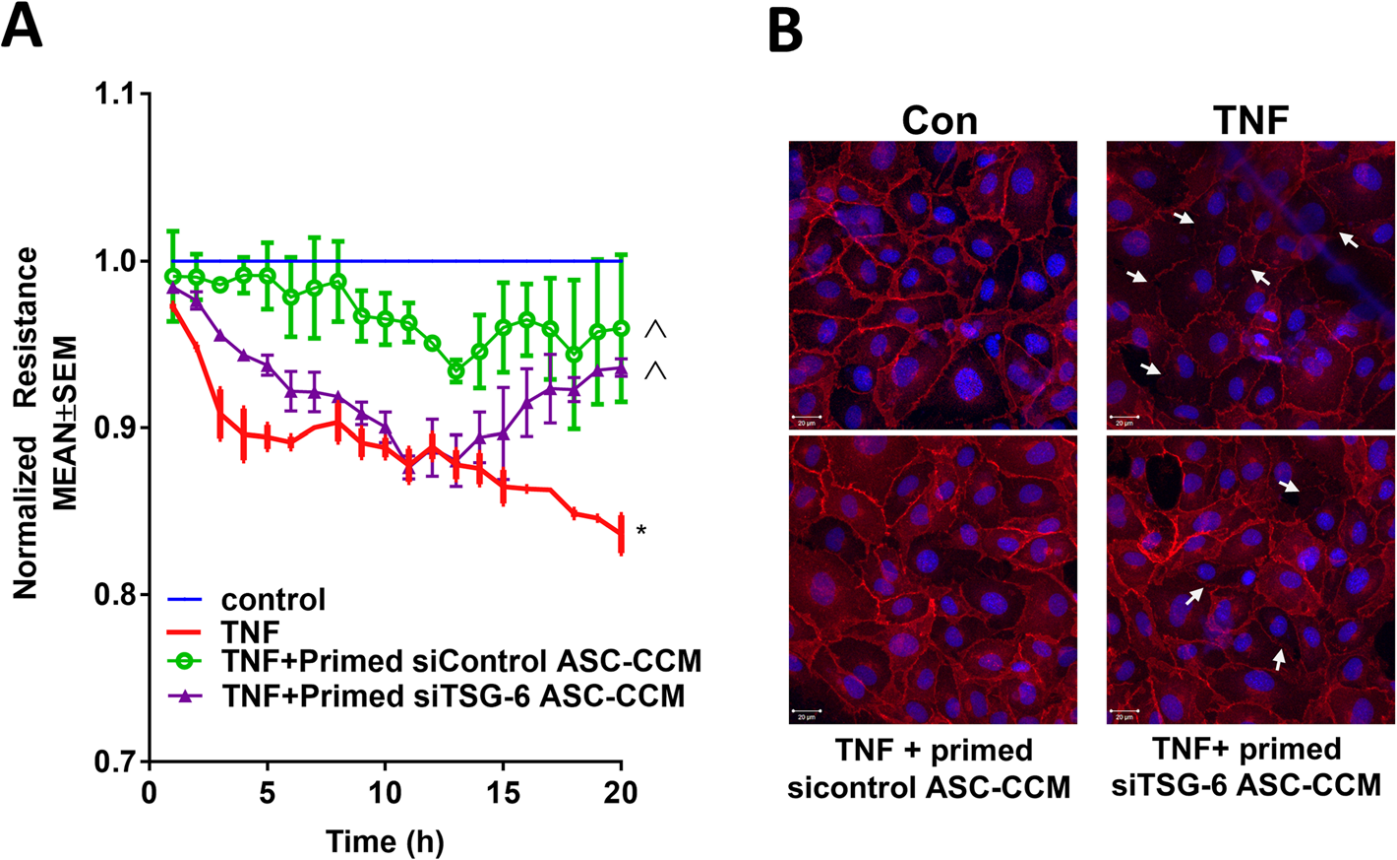
Depletion of TSG-6 from ASC-CCM fails to suppress TNFα-induced trans-endothelial resistance. **a** Trans-endothelial resistance is protected by siControl ASC-CCM, but not by siTSG-6 TSG-6 ASC-CCM in vitro. Representative ECIS tracings plotted as normalized resistance expressed as mean ± SEM of a single experiment performed in replicates with similar data from two independent experiments. *p < 0.001 compared to control, ^*p* < 0.05 compared to TNF. **b** Representative confocal immunofluorescence images of VE-cadherin in HREC. White arrows indicate loss of cell-cell contacts

Previously, we have shown that ASC-CCM could preserve retinal barrier function in an in vitro model [11]. To address the role of TSG-6 in this pathway, trans-endothelial electrical resistance (TER) was measured in human retinal endothelial cells (HRECs) exposed to TNFα. Whereas TNFα-treated HREC demonstrated sustained reduction in the barrier integrity as evidenced by decreased TER at the 20-h time point (TNFα, 0.83 ± 0.01; control, 1.0; *p* < 0.001), only cells pretreated with primed siControl-ASC-CCM, but not primed siTSG-6-ASC-CCM, partially rescued the loss in TER (0.95 ± 0.04, *p* < 0.05, Fig. 3a). Since we found that alterations in TER coincided with a change in VE-cadherin distribution [24], we also assessed VE-cadherin distribution. While control untreated cells displayed a continuous pattern of VE-cadherin staining associated with the lateral cell borders, treatment with TNFα caused a disruption in the pattern of VE-cadherin with punctate staining on the cell surface. On the other hand, HREC treated simultaneously with primed siControl-ASC-CCM and challenged with TNFα demonstrated restoration of VE-cadherin staining similar to control cells. Interestingly, cells pretreated with siTSG-6 ASC-CCM failed to rescue the VE-cadherin distribution when challenged with TNFα (Fig. 3b). Taken together, these findings suggest that depletion of TSG-6 from ASC-CCM fails to protect against the TNFα-induced loss of endothelial barrier integrity.

### TSG-6-depleted ASC-CCM fails to suppress phosphorylated STAT3 in stimulated BV2 and HRECs

**Fig. 4:**
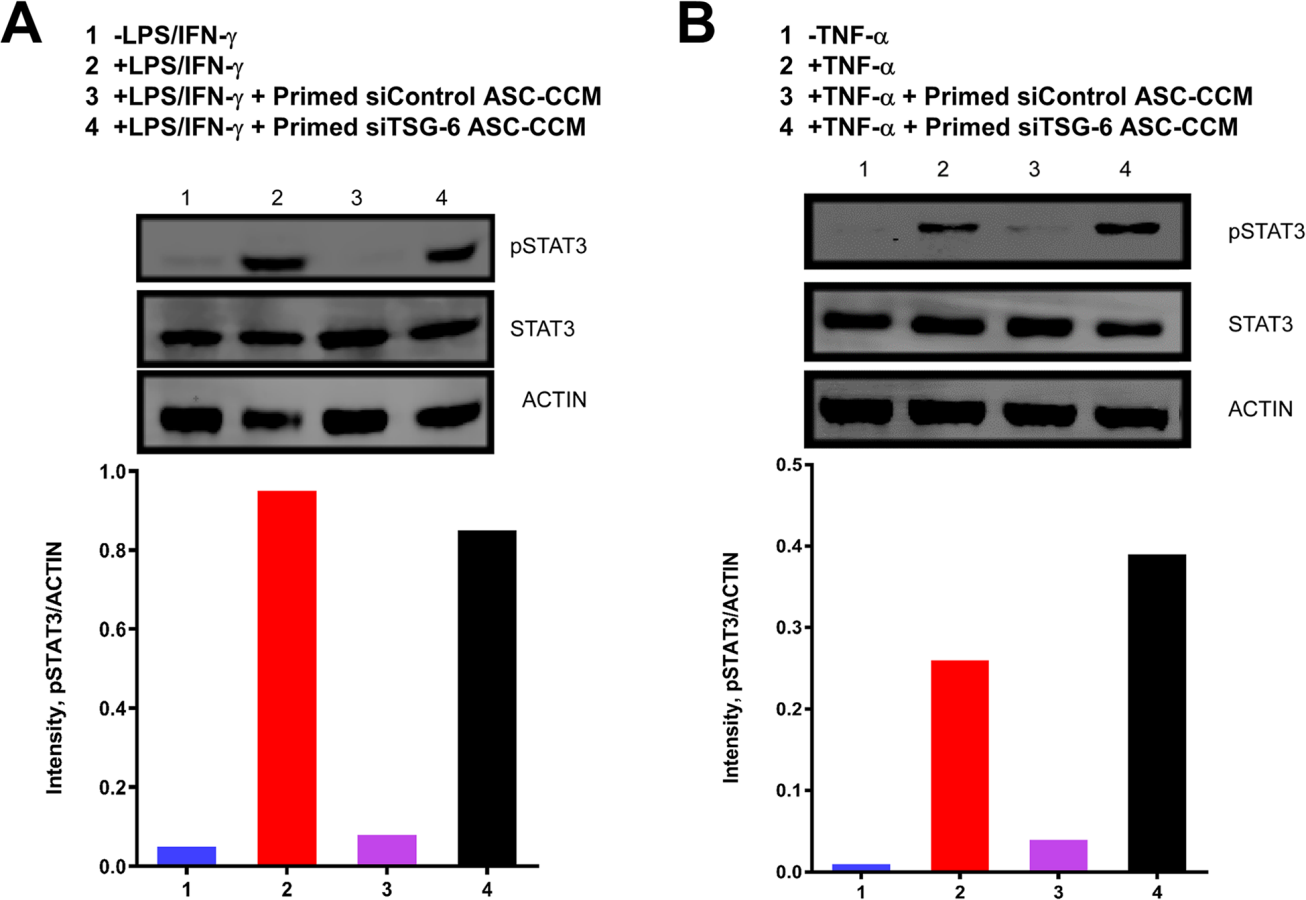
Depletion of TSG-6 from ASC-CCM fails to suppress phosphorylated STAT3 in activated BV2 microglia and human retinal endothelial cells. Immunoblot analysis of total STAT3 and pSTAT3 in **a** BV2 and **b** HREC lysates. Actin served as internal control remained unchanged. Data represent a single experiment with similar data from another independent experiment

Previously, it was shown that TSG-6 regulates the activation of STAT proteins downstream of LPS signaling in *TSG6*^*−/−*^ mice [28]. Since STAT3 has been implicated in promoting inflammatory pathways, we reasoned that TSG-6 through a STAT3 pathway might play an anti-inflammatory role and suppress microglial activation. To this end, we measured total STAT3 and phosphorylated STAT3 in BV2 microglial cells that were activated with LPS and IFN-γ with and without primed siControl-ASC-CCM or primed siTSG-6-ASC-CCM. While primed siControl-ASC-CCM suppressed STAT3 phosphorylation in BV2 cells treated with LPS and IFN-γ, primed siTSG-6-ASC-CCM failed to mitigate STAT3 phosphorylation (Fig. 4a). Because STAT3 is also implicated in endothelial permeability [29], we measured STAT3 levels in HRECs that were activated with TNFα with and without primed siControl-ASC-CCM or primed siTSG-6-ASC-CCM. While primed siControl-ASC-CCM suppressed STAT3 phosphorylation in HRECs treated with TNFα, primed siTSG-6-ASC-CCM failed to mitigate STAT3 phosphorylation (Fig. 4b).

### TSG-6-depleted ASC-CCM fails to suppress visual deficits in blast-induced damage

**Fig. 5:**
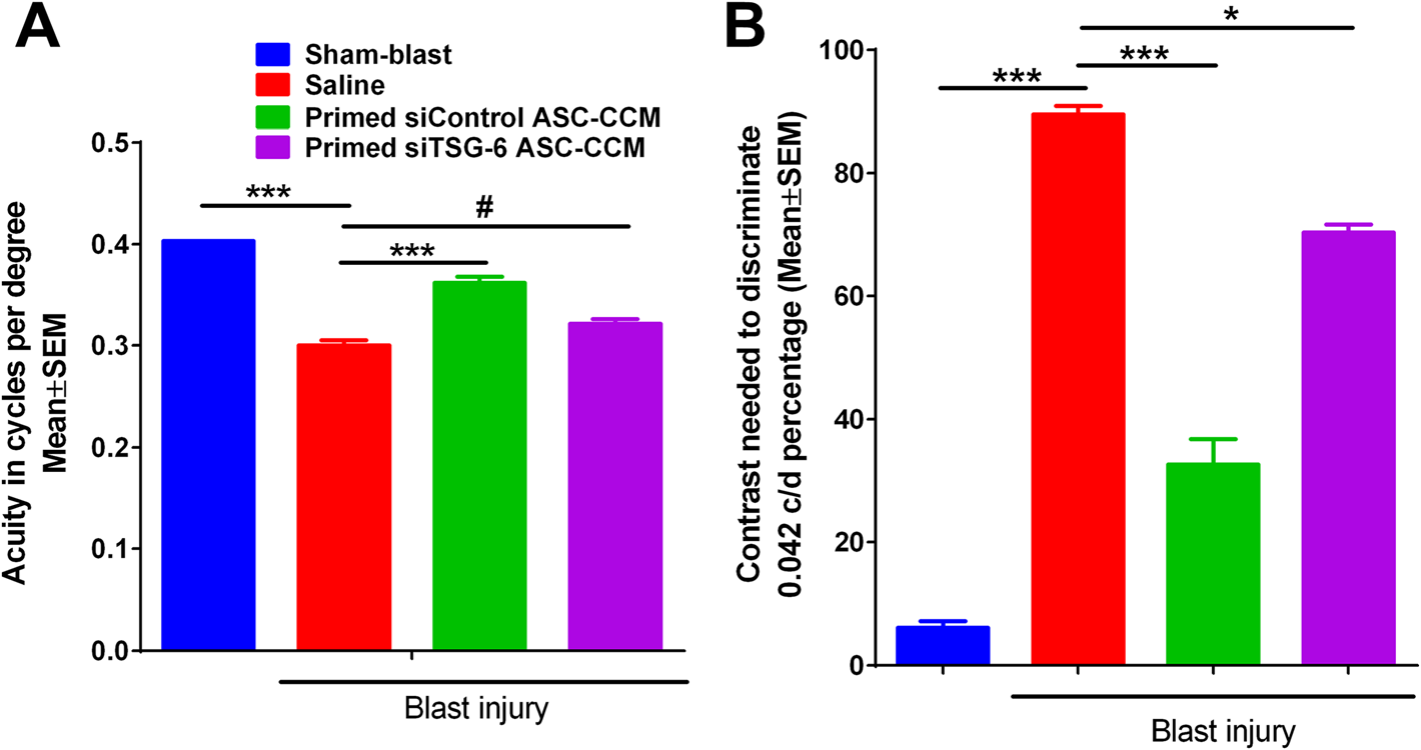
A depletion of TSG-6 from ASC-CCM fails to improve visual acuity and contrast sensitivity in blast injury mice. **a** Visual acuity was measured by presenting black and white bars of varying spatial frequencies at 100% contrast. **b** Contrast sensitivity was measured by changing the contrast gradient that generates tracking at a fixed spatial frequency of 0.042 c/d. Contrast sensitivity in mice is expressed as a percentage, with a higher percent contrast requirement indicating less contrast sensitivity. Data represent mean ± SEM from *n* = 10–20 animals/group. *p < 0.05; ***p < 0.001; ^#^*p* > 0.05

Previously, we have shown that ASC-CCM could preserve visual acuity and contrast sensitivity after blast injury in our mTBI model [11]. To understand the role of TSG-6 in the observed beneficial effects, we performed optokinetic measurements and electroretinogram measurements 4 weeks after blast injury in the sham blast group that received saline, 50-psi blast group that received saline, 50-psi blast group that received primed siControl-ASC-CCM, and 50-psi blast group that received primed siTSG-6-ASC-CCM. While sham blast mice had a visual acuity of 0.403 ± 0.00 c/d in the left eye, visual acuity in blast mice was significantly decreased in the left eye (0.300 ± 0.005; sham, 0.406 ± 0.01 c/d, *p* < 0.001). Interestingly, mice that received primed siControl-ASC-CCM, but not primed siTSG-6-ASC-CCM, demonstrated a significant improvement in visual acuity (primed siControl-ASC-CCM, 0.362 ± 0.006 c/d; primed siTSG-6-ASC-CCM, 0.321 ± 0.004 c/d, *p* < 0.001, Fig. 5a). Similarly, the contrast sensitivity of blast mice showed an increase in the contrast needed to detect 0.042 c/d in the left eye (blast, 89.54% ± 1.35; sham, 6.08% ± 1.08, *p* < 0.001), with a significant improvement observed in mice receiving only primed siControl-ASC-CCM but not primed siTSG-6-ASC-CCM in the left eye (primed siControl-ASC-CCM, 32.68% ± 4.11; primed siTSG-6-ASC-CCM, 70.03% ± 1.29, *p* < 0.001; Fig. 5b).

ERG is one of the most widely used measures of functional performance/deficits in the visual pathway in diabetic rodents [30, 31]. Dark-adapted scotopic ERG responses were recorded from both eyes of all study groups. With increasing flashlight intensities starting from 0.0025 to 25 cd s m^2^, an expected increase in amplitudes could be discerned with the most robust changes detected at 25 cd s m^2^ in sham blast mice. The b-wave amplitude at 25 cd s m^2^ flash light intensity in the sham blast group of animals were 455.3125 ± 8.8 μV which significantly decreased to 410.888 ± 13.08 μV in blast injury mice that received saline (*p* < 0.007, Additional file 4: Figure S4). On the other hand, intravitreal injection of both primed siControl-ASC-CCM and primed siTSG-6-ASC-CCM resulted in improvement in b-wave amplitude at 25 cd s m^2^, 447.7 ± 16.13 μV for the blast-primed siControl-ASC-CCM group (*p* < 0.04), and 469.09 ± 18.64 μV for the blast-primed siTSG-6-ASC-CCM group (Additional file 4: Figure S4; *p* < 0.008).

### TSG-6-depleted ASC-CCM fails to reduce blast-induced expression of the glial fibrillary acidic protein (GFAP) in Müller cells

**Fig. 6:**
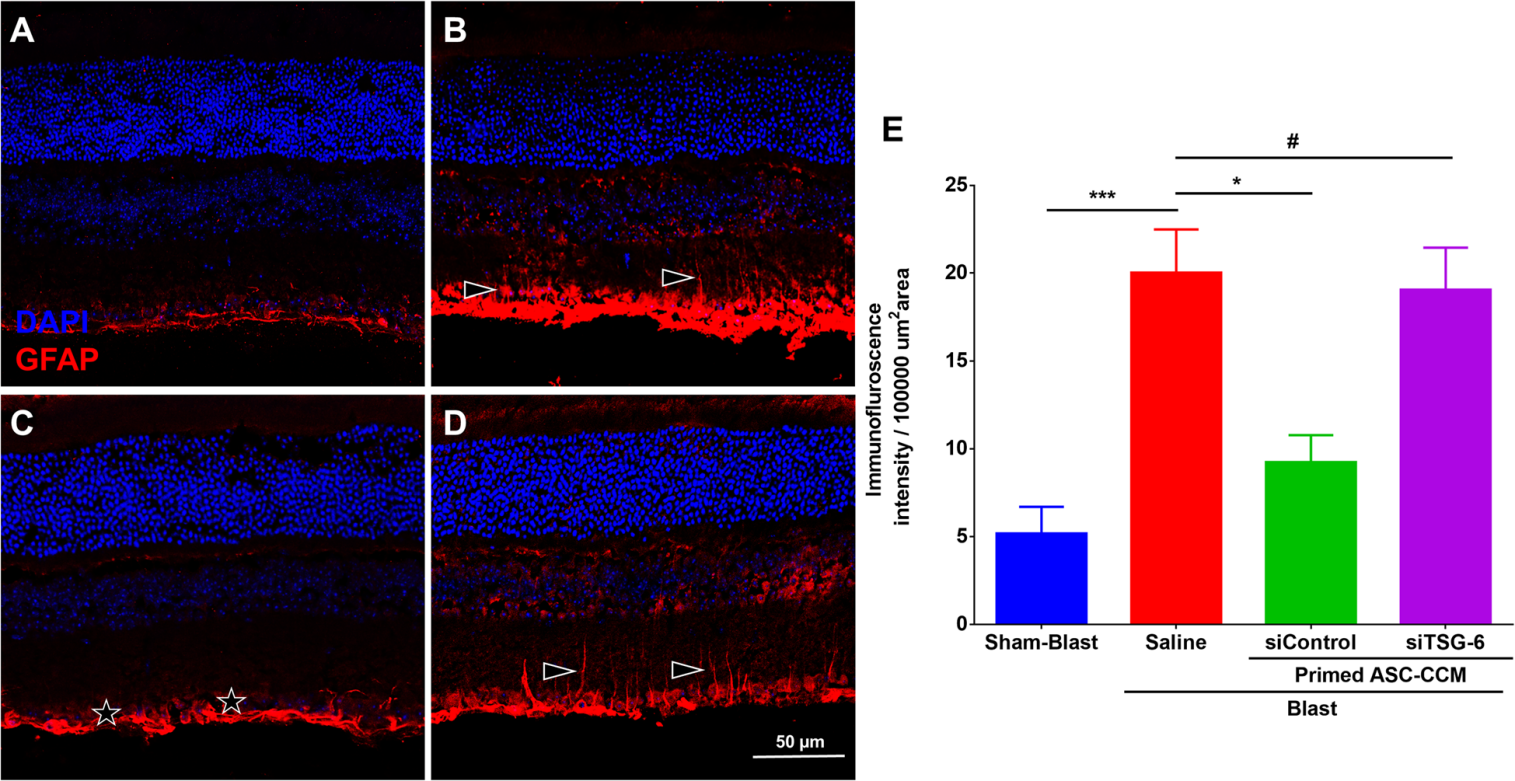
Depletion of TSG-6 from ASC-CCM fails to improve retinal GFAP expression in blast injury mice. GFAP immunoreactivity in the retina of **a** sham and **b** blast injury animals that received saline, **c** blast injury animals that received primed siControl ASC-CCM, and **d** blast injury animals that received siTSG-6 ASC-CCM. **e** ImageJ quantification of GFAP intensity. Data represent mean ± SEM from *n* = 5–9 animals/group. *p < 0.01; ***p < 0.001. Scale bar = 50 μm

Previously, we have shown that ASC-CCM could suppress increased GFAP expression in Müller glial cells in blast injury mouse retina [11]. To address the role of TSG-6 in the observed beneficial effects, we assessed GFAP immunoreactivity in all experimental groups. While in the blast group with saline, GFAP expression was found in the Muller glial processes that extended into the inner retina, only blast mice that received primed siControl-ASC-CCM but not the primed siTSG-6-ASC-CCM demonstrated lower levels of GFAP expression (Fig. 6). The mean total pixel intensity of GFAP expression measured from NFL to retinal pigment epithelium in the normal sham group retina was 5.18 ± 1.5 while the blast group with saline was 20.02 ± 2.46 (mean intensity/100,000 μm^2^ area; *p* < 0.002, *n* = 5; Fig. 6e). On the other hand, blast mice with primed-siControl-ASC-CCM showed reduced GFAP expression (9.24 ± 1.53 mean intensity/100,000 μm^2^ area; *p* < 0.01, *n* = 9) whereas blast mice with primed-siTSG-6-ASC-CCM remained high (19.05 ± 2.39 mean intensity/100,000 μm^2^ area; *p* > 0.01, *n* = 9).

### TSG-6-depleted ASC-CCM fails to suppress pro-inflammatory gene transcripts in blast mice retina

**Fig. 7:**
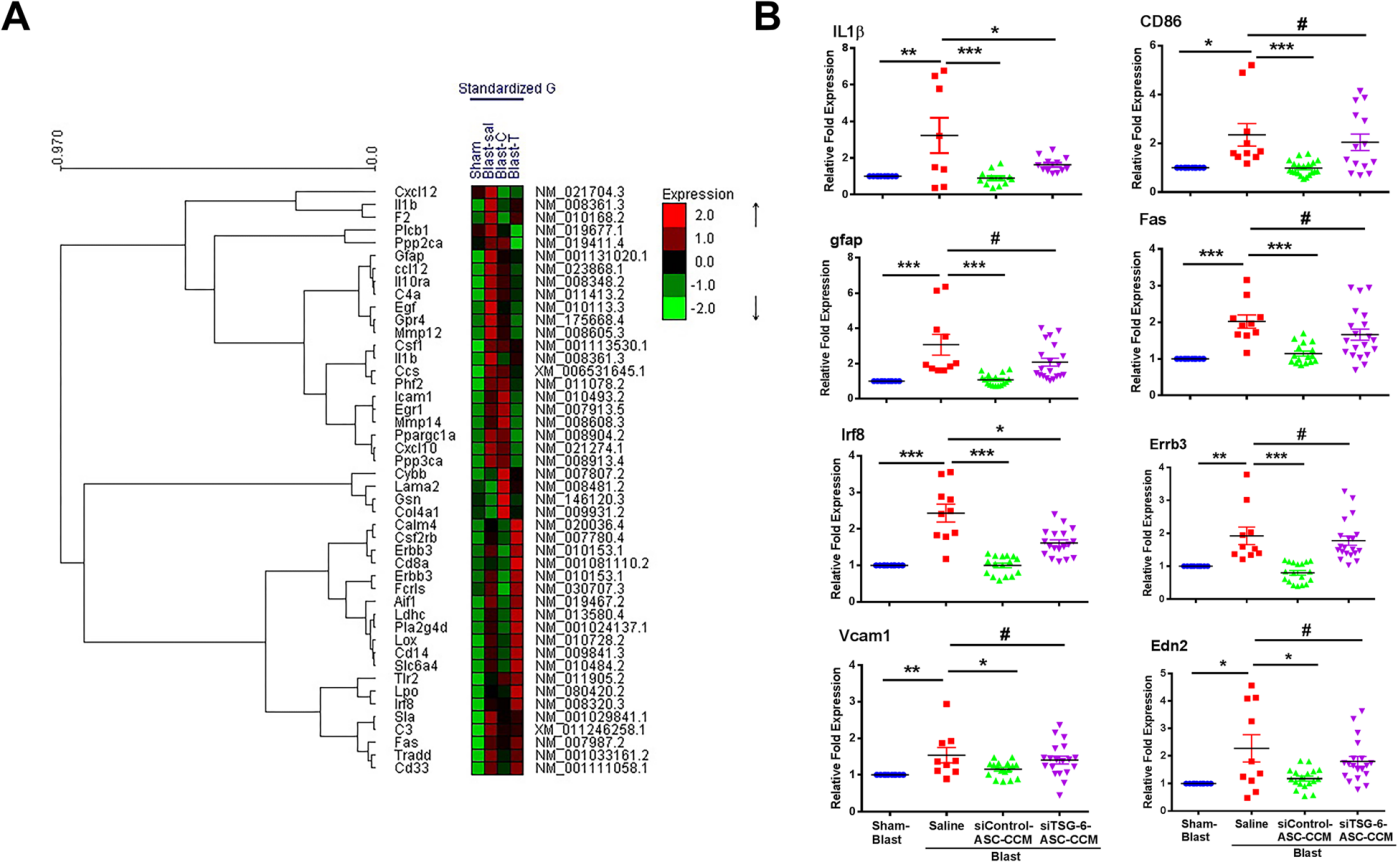
Depletion of TSG-6 from ASC-CCM fails to improve inflammatory gene expression in the retina of blast injury mice. **a** The RNA was isolated and analyzed by NanoString neuropathology gene panel. Heat map showing hierarchical clustering of genes upregulated (red) or downregulated (green) in retinal tissues from sham blast mice (sham), blast injury mice that received intravitreal saline (Blast-sal), blast injury mice that received primed siControl ASC-CCM (Blast-C), and blast injury mice that received primed siTSG-6-ASC-CCM (Blast-T) (*n* = 4–9 per group). **b** TaqMan gene expression analysis of representative genes selected from NanoString multiplex RNA analysis from all study groups (*n* = 4–9 per group). *p < 0.05; **p < 0.01; ***p < 0.001; ^#^*p* > 0.05

Previously, we demonstrated the anti-inflammatory capacity of ASC-CCM in suppressing the expression of Il1β and CD86 (early and late markers of the M1 phenotype of microglia) 4 weeks after blast injury [11]. In this study, to address the role of TSG-6 in the observed effects, retinal extracts from blast mice from all groups were assessed for gene expression by NanoString multiplex analysis followed by TaqMan probe-based real-time PCR analyses. The heat maps (Fig. 7a; Additional file 5: Figure S5A) and volcano plots (Additional file 5: Figure S5B) show several interesting comparisons between the sham blast and blast injury animals that received intravitreal saline injections. A number of genes that are involved in neuroinflammation, neurotransmission, metabolism, neuroplasticity, development and aging, and neuron-glia interactions are increased > 2-fold in blast injury compared to sham blast. Among these, genes involved in neuroinflammation (C3, complement factor 3; Tlr2, Toll-like receptor 2; Irf8, interferon regulatory factor 8; Fas, TNF receptor superfamily member 6; Lox, lysyl oxidase) are increased in blast injury but were decreased in blast animals that received siControl ASC-CCM but not siTSG-6 ASC-CCM. Similarly, genes involved in neurotransmission (Erbb3, erb-b2 receptor tyrosine kinase 3; Slc6a4, solute carrier family 6 (neurotransmitter transporter, serotonin), member 4) are increased in blast injury but were decreased in blast animals that received primed siControl ASC-CCM but not by primed siTSG-6 ASC-CCM (Additional file 5: Figure S5C).

To confirm these observations, individual TaqMan assays for representative genes were performed as we described previously [11]. Figure 7b shows a representative scatter plot data of individual genes in all four groups of mice. Blast injury mice receiving saline had significantly increased abundance of gene transcripts involved in microglial activation (Il1β, CD86, Gfap), endothelial activation (Vcam1, Edn2), neuroinflammation (Fas, Irf8), and neurotransmission (Erbb3) compared to sham mice. Interestingly, four out of the six genes assessed were significantly changed in blast mice that received primed siControl-ASC-CCM but not the primed siTSG-6-ASC-CCM. The normalized fold change in the expression of Il1β (blast vs blast/primed-siControl-ASC-CCM, 3.24 ± 0.96 vs 0.89 ± 0.09, *p* < 0.001; blast vs blast/primed-siTSG-6-ASC-CCM, 3.24 ± 0.96 vs 1.63 ± 0.09, *p* < 0.037), CD86 (blast vs blast/primed-siControl-ASC-CCM, 2.35 ± 0.51 vs 0.98 ± 0.07, *p* < 0.002; blast vs blast/primed-siTSG-6-ASC-CCM, 2.35 ± 0.51 vs 2.04 ± 0.33, *p* > 0.05), Gfap (blast-saline vs blast/primed-siControl-ASC-CCM, 3.07 ± 0.58 vs 1.07 ± 0.08, *p* < 0.001; blast-saline vs blast/primed-siTSG-6-ASC-CCM, 3.07 ± 0.58 vs 2.08 ± 0.22, *p* > 0.05), Fas (blast-saline vs blast/primed-siControl-ASC-CCM, 2.02 ± 0.18 vs 1.14 ± 0.02, *p* < 0.001; blast-saline vs blast/primed-siTSG-6-ASC-CCM, 2.02 ± 0.18 vs 1.66 ± 0.15, *p* > 0.05), Irf8 ((blast-saline vs blast/primed-siControl-ASC-CCM, 2.4 ± 0.24 vs 1.001 ± 0.06, *p* < 0.001; blast-saline vs blast/primed-siTSG-6-ASC-CCM, 2.4 ± 0.24 vs 1.61 ± 0.08, *p* < 0.01), Erbb3 (blast-saline vs blast/primed-siControl-ASC-CCM, 1.92 ± 0.26 vs 0.79 ± 0.06, *p* < 0.003; blast-saline vs blast/primed-siTSG-6-ASC-CCM, 1.92 ± 0.26 vs 1.77 ± 0.13, *p* > 0.05), Vcam1 (blast-saline vs blast/primed-siControl-ASC-CCM, 1.76 ± 0.27 vs 1.001 ± 0.06, *p* < 0.001; blast-saline vs blast/primed-siTSG-6-ASC-CCM, 1.76 ± 0.27 vs 1.53 ± 0.16, *p* < 0.01), and End2 (blast-saline vs blast/primed-siControl-ASC-CCM, 2.27 ± 0.49 vs 1.17 ± 0.03, *p* < 0.003; blast-saline vs blast/primed-siTSG-6-ASC-CCM, 2.27 ± 0.49 vs 1.90 ± 0.19, *p* > 0.05) gene transcripts is shown in Fig. 7b.

## Discussion

**Fig. 8:**
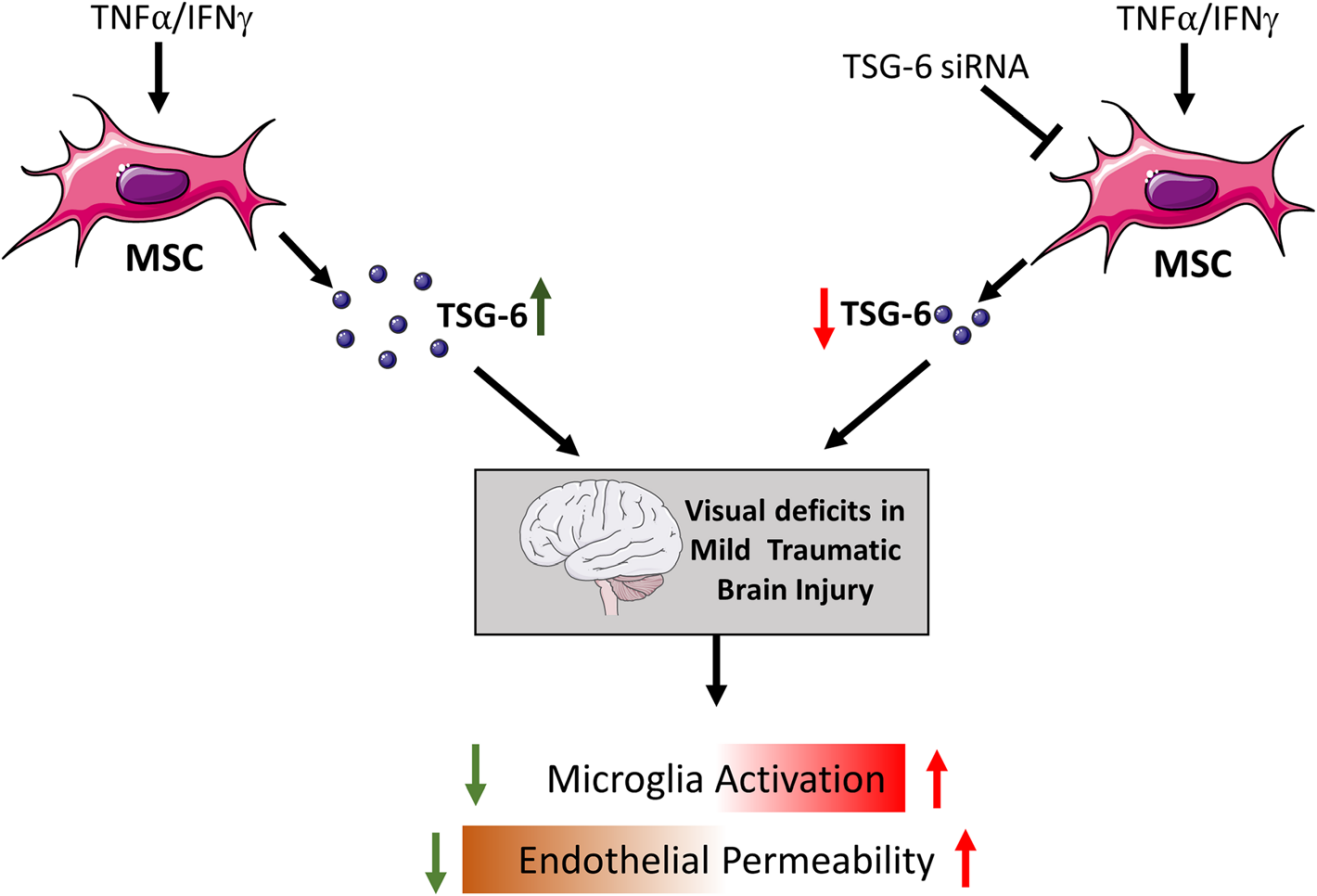
Schematic model of TSG-6 in conditioned media from adipose mesenchymal stem cells protects against visual deficits in mild traumatic brain injury model through neurovascular modulation. Direct effects of TSG-6 on multiple cell types within the retina in establishing a cell signaling environment that protects visual processing from the effects of acute blast injury via decreased microglial and endothelial activation and protection against loss of endothelial permeability. Created with content from Servier Medical Art (https://smart.servier.com) under Creative Commons Attribution 3.0 Unported license (https://creativecommons.org/licenses/by/3.0/)

Our previous published study demonstrated the beneficial effects of concentrated conditioned medium from cytokine-primed ASC in the mTBI mouse model. It was demonstrated that treatment prevented the activation of microglia cells after an injury that likely contributed to the overall prevention of vision loss in these animals. It was also observed that TSG-6 is upregulated in ASC-CCM by about fivefold upon stimulation with TNFα and IFNγ with no changes in TIMP1 or COXIV, suggesting a pivotal role for TSG-6 in modulating inflammation and the injury response [11]. The present study provides substantial evidence that microglial activation is significantly prevented via TSG-6. When we removed TSG-6 from the ASC-CCM, the beneficial effects of treatment in the mTBI model were attenuated. Interestingly, we observed that the role of TSG-6 is not limited to suppression of microglial activation but likely extends to the protection against loss of endothelial barrier integrity based on our in vitro findings. In support of our data, several other studies have shown the beneficial effects of TSG-6 secreted by ASCs or MSCs in disease models such as severe acute pancreatitis [17], acute lung injury [32], corneal injury [20], asthma [19], diabetic corneal epithelial wound healing [18], myocardial infarction [33], and brain injury [16, 34]. Taken together, our study supports a positive regulatory role for TSG-6 released by ASCs in the amelioration of visual deficits of mTBI. Most importantly, this work broadens our understanding of the direct effects of TSG-6 on multiple cell types within the retina in establishing a cell signaling environment that protects visual processing from the effects of acute blast injury (Fig. 8).

TSG-6 is a 30-kDa anti-inflammatory protein expressed by MSCs but also monocytes and macrophages [12]. A variety of pro-inflammatory stimuli have been shown to upregulate TSG-6 that include TNFα, IL1β, and LPS [35]. Although the exact mechanism through which TSG-6 elicits its anti-inflammatory effects is not fully understood, it is known to polarize macrophages from a pro-inflammatory to anti-inflammatory phenotype in a STAT1 and STAT3 dependent pathway [28]. To this end, BV2 microglia treated with ASC-CCM that lacks TSG-6 failed to suppress nitrite release, pro-inflammatory gene expression, and STAT3 phosphorylation after LPS stimulation, suggesting a key role for TSG-6 in microglial activation released by ASCs. Furthermore, both arborization of microglial cells as assessed by F-actin staining and increased Iba-1 immunostaining in BV2 cells were abrogated by siControl ASC-CCM but not the siTSG-6-ASC-CCM. The microglia have the capacity to shift a cell-to-cell signaling microenvironment from neurotoxic to neuroprotective depending on the type of injury, tissue location, and the timing of assessment. Consequently, we utilized NanoString gene expression analysis to better understand how treatment altered the injury environment via changes in microglial-related genes. The analysis revealed that more than 20 genes were upregulated in blast injury animals 4 weeks post-blast injury. For example, interferon response pathway genes such as the Irf8 transcription factor, Ccl5 and Cxcl10, and TNF signaling genes such as Tnf and Il1β were upregulated by at least twofold in blast injury retina, while they were found to be downregulated in the siControl ASC-CCM-treated retina. Interestingly, our TaqMan assays for both Irf8 and Il1b confirmed this differential expression and a significant rescue with siControl ASC-CCM, which is in line with the association of these genes with microglia and mTBI [36, 37]. Collectively, these studies validate the hypothesis that TSG-6 is required for the beneficial outcomes of ASC-CCM on microglia. These studies indicate that TSG-6 in ASC-CCM is required for the observed outcomes; however, considering the fact that some microglial-associated gene expression was also altered with siTSG-6-ASC-CCM, TSG-6 alone may be insufficient to recapitulate the full breadth of the observed therapeutic effect.

Several anti-inflammatory agents have been used in animal models with limited benefits with some used in clinical trials of TBI failed [38–41]. Acute exposure of TSG-6 has been shown to prevent neurodegeneration in a TBI animal model resulting in improved long-term memory and behavioral disability [42]. In accordance with this finding, our study has shown a neuroprotective role of TSG-6 released by ASC-CCM in the rescue of visual deficits in our mTBI model. Only the siControl-ASC-CCM but not siTSG-6-ASC-CCM improved visual acuity and contrast sensitivity in the mice. These observations are also in agreement with our previous observations of improvement in optokinetic measurements in TBI mice [11]. Additionally, ERG analysis demonstrated amelioration of b-wave, a bipolar response that is suppressed in blast injury retina with siControl-ASC-CCM. Interestingly, animals that received siTSG-6-ASC-CCM also showed improvement, suggesting molecules other than TSG-6 in ASC-CCM affect the bipolar response. On the other hand, since the siControl-ASC-CCM but not siTSG-6-ASC-CCM reduced GFAP expression in the inner retina, TSG-6 appears to play a role in preventing of Muller cell gliosis. Given the critical supportive role of Muller cells to retinal ganglion cells, normalization of Muller cell phenotype and function may result in improved function of retinal ganglion cells and the processing of visual signals to the optic nerve. Increased distribution of GFAP throughout Muller glia is a common feature of a variety of retinal diseases, and correlates with neuronal degeneration and loss, resulting in retinal thinning, observed in animal models [43]. We previously observed a focal loss of neuronal cells in the GCL of blast retina [11]. Taken together, our data suggest that the trophic factors specifically TSG-6 from ASCs may rescue ganglion cell or other neuronal cell damage in the blast retina indirectly via the Muller cells.

A growing number of neurological diseases are characterized by a highly vascular phenotype. Considering the fact that TBI is represented with a compromised blood-brain barrier [44], we anticipated the loss of the endothelial barrier in our mTBI model. Therefore, we tested endothelial cell permeability stimulated with TNFα in the absence of immune cells and observed a critical role for TSG-6 in normalizing permeability mainly by preserving the endothelial adherens junctions as observed with VE-cadherin immunostaining and downregulation of STAT3 phosphorylation. Consequently, the role of TSG-6 extends beyond shifting microglial and/or macrophage polarization observed in other studies and may serve as an important regenerative agent to block pro-inflammatory signaling initiated by immune cells on the endothelial cells recruited to the sites of blast injury. Future studies will aim to understand if TSG-6 in ASC-CCM could protect against increased vascular permeability in vivo.

It has been shown that TSG-6 acts via the STAT3 pathway [28]. Various cytokines including IFNγ, growth factors, and G-protein coupled receptors induce STAT3 phosphorylation [45, 46], demonstrating that diverse pathways lead to STAT3 activation. Upon its activation, STAT3 subsequently translocates into the nucleus where it stimulates the transcription of genes, resulting in either anti-inflammatory effects [47] or pro-inflammatory effects [48]. Specifically in retinal inflammation, activated STAT3 expression is increased in the ganglion cell, inner nuclear, and photoreceptors layers, therefore linking STAT3 to a pro-inflammatory effect and subsequent excessive degradation of proteins required for visual function [49]. In support of our observation that ASCs respond to pro-inflammatory cytokines by secreting a higher concentration of TSG-6 that suppresses pro-inflammatory microglial and endothelial activation in a STAT3 dependent pathway, human MSCs are found to be neuroprotective in optic neuropathies through suppression of STAT3 and other pathways [50]. More studies are warranted to better understand the regulation of STAT3 in our mTBI model and its link to neurovascular degeneration.

Since TSG-6 expression in ASC-CCM mediates many of the observed therapeutic benefits of ASC-CCM in our model, direct administration of TSG-6 may likely have therapeutic activity. In support of this hypothesis, a recent study suggested that acute administration of TSG-6 showed remarkable improvement in memory after TBI [42]. However, it must be noted that TSG-6 was effective only when administered during the initial and mild phase of an inflammatory reaction in a corneal mechanical injury model [20] with no therapeutic benefit in a corneal alkali injury model [51]. This suggests that TSG-6 alone may not likely to prevent ongoing tissue degeneration in TBI, while on the other hand, the powerful antioxidant and immunomodulatory proteins in addition to TSG-6 in ASC-CCM will not only suppress ongoing inflammation but also aid in the regeneration.

## Conclusions

In conclusion, our findings suggest that ASCs respond to an inflammatory milieu by secreting several therapeutically valuable proteins among which TSG-6 expression correlated with the therapeutic potency of ASC-CCM. ASCs engineered to produce TSG-6 will be an invaluable regenerative therapy solution against the traumatic effects of blast injuries to the retina with potential broader applications to other degenerative central nervous system diseases. Alternatively, processes to improve the production of key analytes such as TSG-6 in ASCs may serve to develop better secretome-derived biologics for the treatment against the traumatic effects of blast injuries to the retina as well as other inflammatory conditions.

## Supplementary information

**Additional file 1: Figure S1.** Experimental time line. About 12-week old C57Bl/6j mice were used in the study. Two independent experiments were performed with the same donor-derived ASC-CM. Live retinal function experiments were performed after 4 weeks of intravitreal injection followed by euthanasia and molecular and histological analyses.

**Additional file 2: Figure S2.** Depletion of TSG-6 from cytokine primed ASC conditioned medium is target specific. Immunoblot analysis of TSG-6 in cell lysates and CCM. GAPDH and TIMP1 in CCM remained unchanged. Data represent a single experiment.

**Additional file 3: Figure S3.** Depletion of TSG-6 from ASC-CCM fails to improve microglial morphology**.** Microglial morphology after the LPS (100 ng/ml) and IFNγ (10 ng/ml) exposure and challenged with siControl ASC-CCM or siTSG-6 ASC-CCM as shown by F-actin stained confocal micrographs. Scale bars = 20 μm. Data represent a single experiment performed in duplicates.

**Additional file 4: Figure S4.** Depletion of TSG-6 from ASC-CCM improves retinal function and vision in blast injury mice. **(A)**: b-wave amplitude measurement in mice at various flash intensities **(B)**: b-wave amplitude at 25 cd.s.cm^2^ expressed as μV. Data represent combined Mean ± SEM from *n* = 8–19 animals/group of the left eye only performed in 2 separate batches. *p < 0.05; **p < 0.01; ***, *p* < 0.001.

**Additional file 5: Figure S5.** Differential gene expression in blast injury mice without and with primed siControl-ASC-CCM and primed siTSG-6-ASC-CCM. **(A):** Heatmap representing the directed global significance score for the represented group comparisons calculated as the square root of the mean signed squared t-statistic for the genes in a gene set, with t-statistics coming from the linear regression underlying the differential expression analysis. **(B)**: Volcano plot displaying each gene’s -log10 (*p*-value) and log2 fold change with the selected covariate. Highly statistically significant genes fall at the top of the plot above the horizontal lines, and highly differentially expressed genes fall to either side. Horizontal lines indicate various False Discovery Rate (FDR) thresholds or p-value thresholds if there is no adjustment to the *p*-values. Genes are colored if the resulting p-value is below the given FDR or p-value threshold. The 40 most statistically significant genes are labeled in the plot. **(C)**: Representative violin plots of differentially expressed genes in all four study groups. **(D)**: Venn diagram showing significant differentially expressed genes. Data represent combined averages from *n* = 4–9 animals/group using NanoString Advanced Analysis.

## Data Availability

All data generated and/or analyzed during this study are included in this published article. Data sharing is not applicable to this article as no datasets were generated or analyzed during the current study. However, the data that support the findings of this study including details of NanoString gene expression analysis are available from the corresponding author upon reasonable request.
