# Supplementary figures and images for: TSG-6 in conditioned media from adipose mesenchymal stem cells protects against visual deficits in mild traumatic brain injury model through neurovascular modulation
Source: Stem Cell Res Ther. 2019 Nov 5;10:318. doi: 10.1186/s13287-019-1436-1 (PMC6833275; doi:10.1186/s13287-019-1436-1)

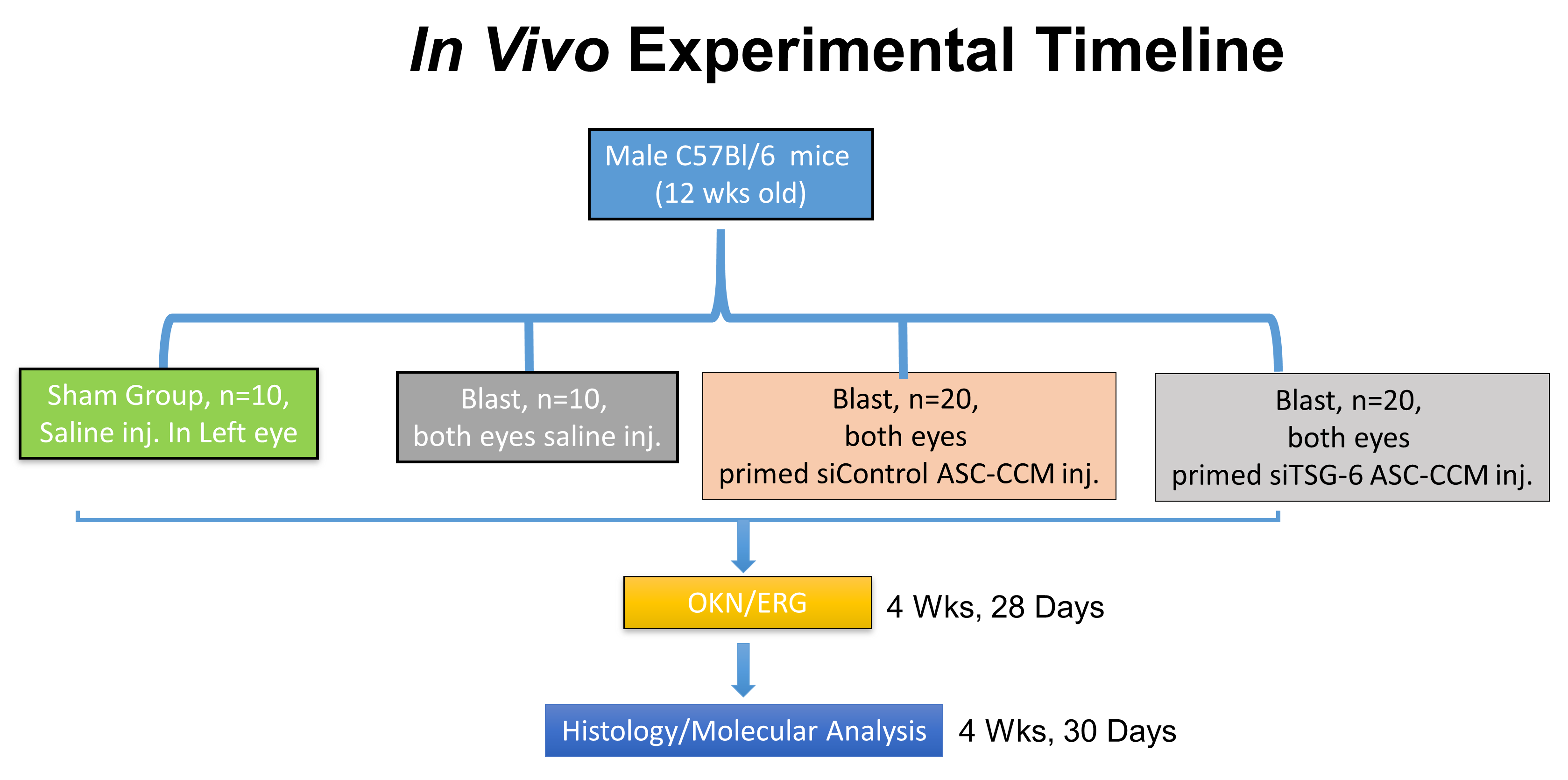

Supplement: Supplementary file 1 — Additional file 1: Figure S1. Experimental time line. About 12-week old C57Bl/6j mice were used in the study. Two independent experiments were performed with the same donor-derived ASC-CM. Live retinal function experiments were performed after 4 weeks of intravitreal injection followed by euthanasia and molecular and histological analyses. [file 13287_2019_1436_MOESM1_ESM.tif]

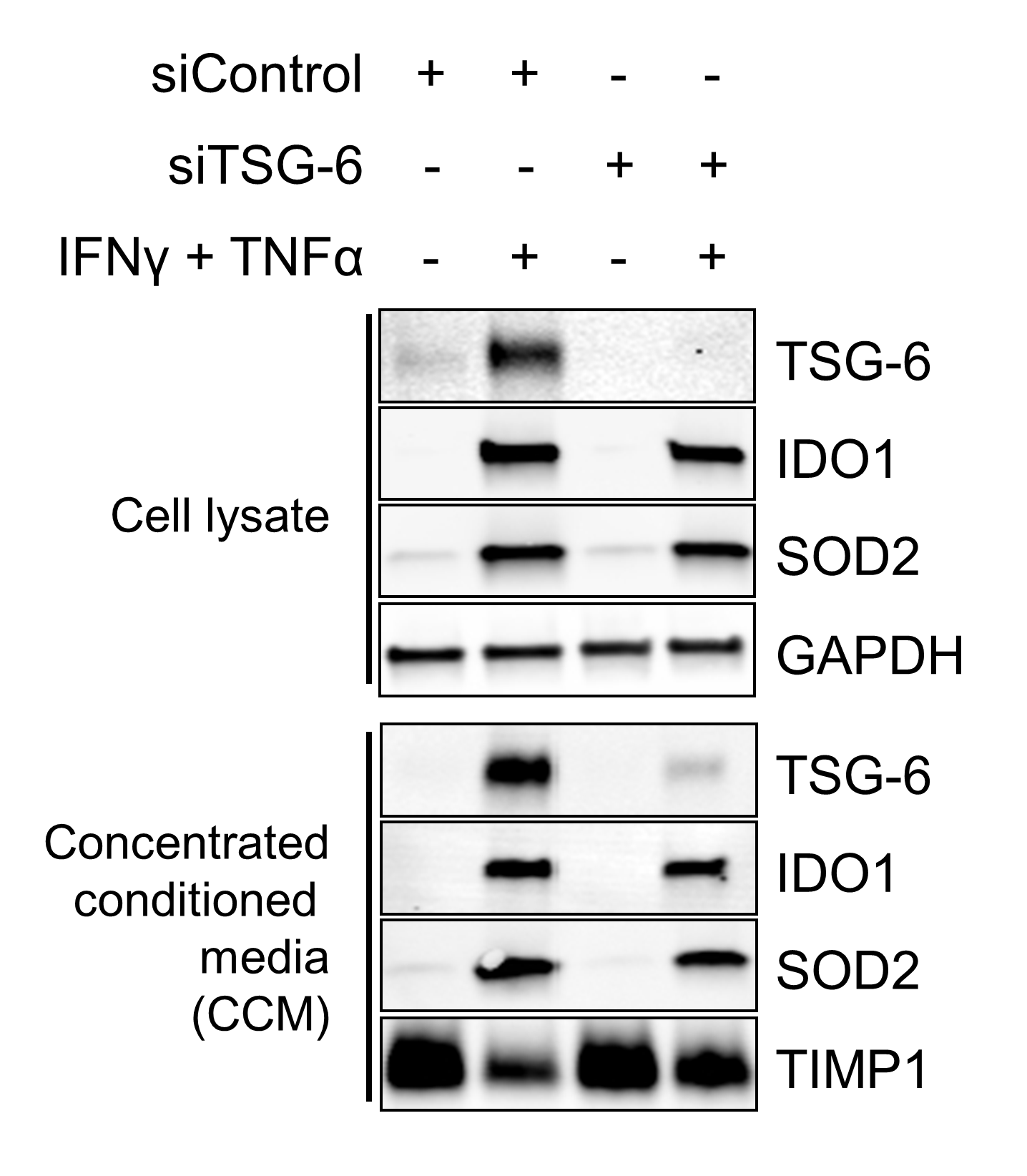

Supplement: Supplementary file 2 — Additional file 2: Figure S2. Depletion of TSG-6 from cytokine primed ASC conditioned medium is target specific. Immunoblot analysis of TSG-6 in cell lysates and CCM. GAPDH and TIMP1 in CCM remained unchanged. Data represent a single experiment. [file 13287_2019_1436_MOESM2_ESM.tif]

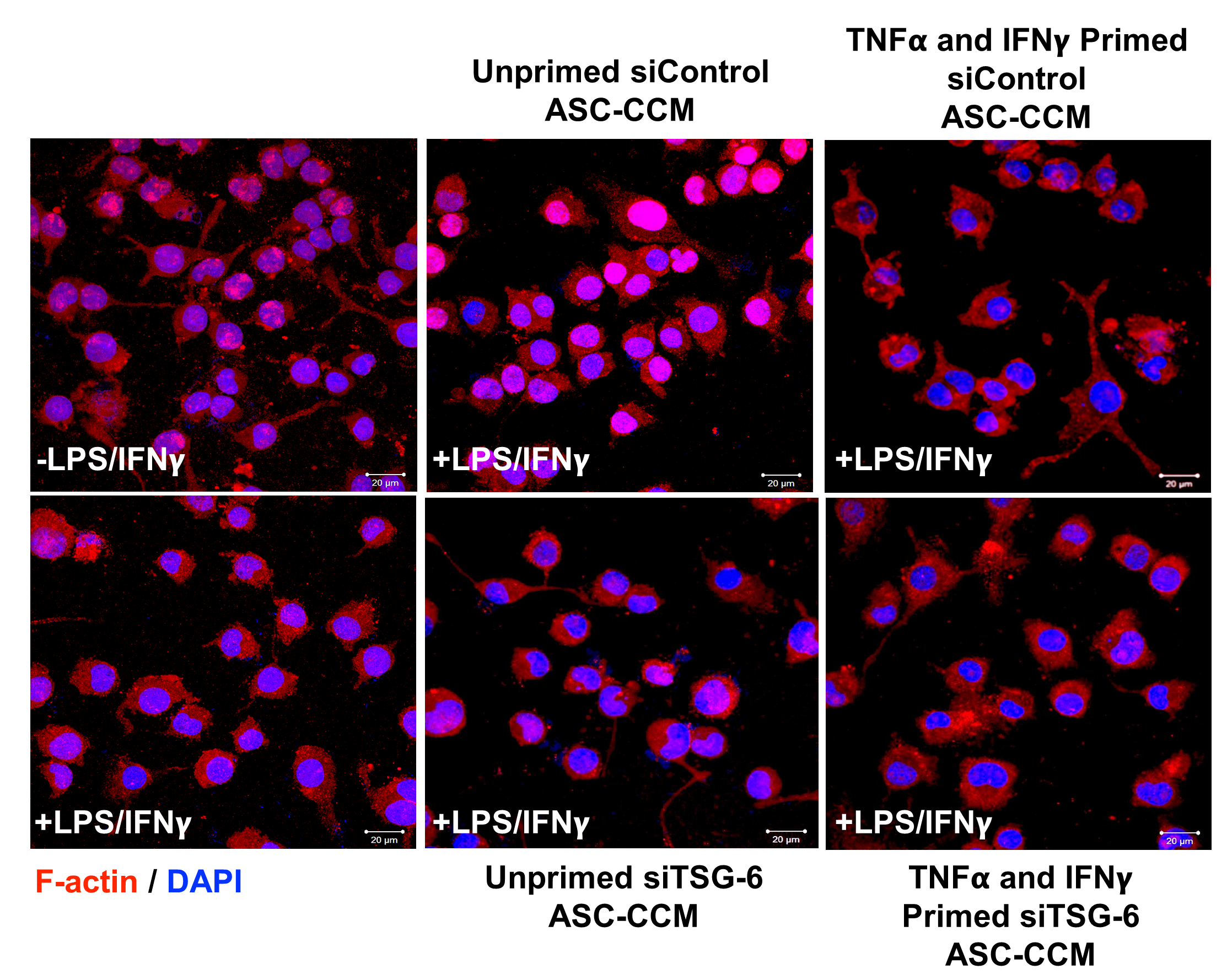

Supplement: Supplementary file 3 — Additional file 3: Figure S3. Depletion of TSG-6 from ASC-CCM fails to improve microglial morphology. Microglial morphology after the LPS (100 ng/ml) and IFNγ (10 ng/ml) exposure and challenged with siControl ASC-CCM or siTSG-6 ASC-CCM as shown by F-actin stained confocal micrographs. Scale bars = 20 μm. Data represent a single experiment performed in duplicates. [file 13287_2019_1436_MOESM3_ESM.tif]

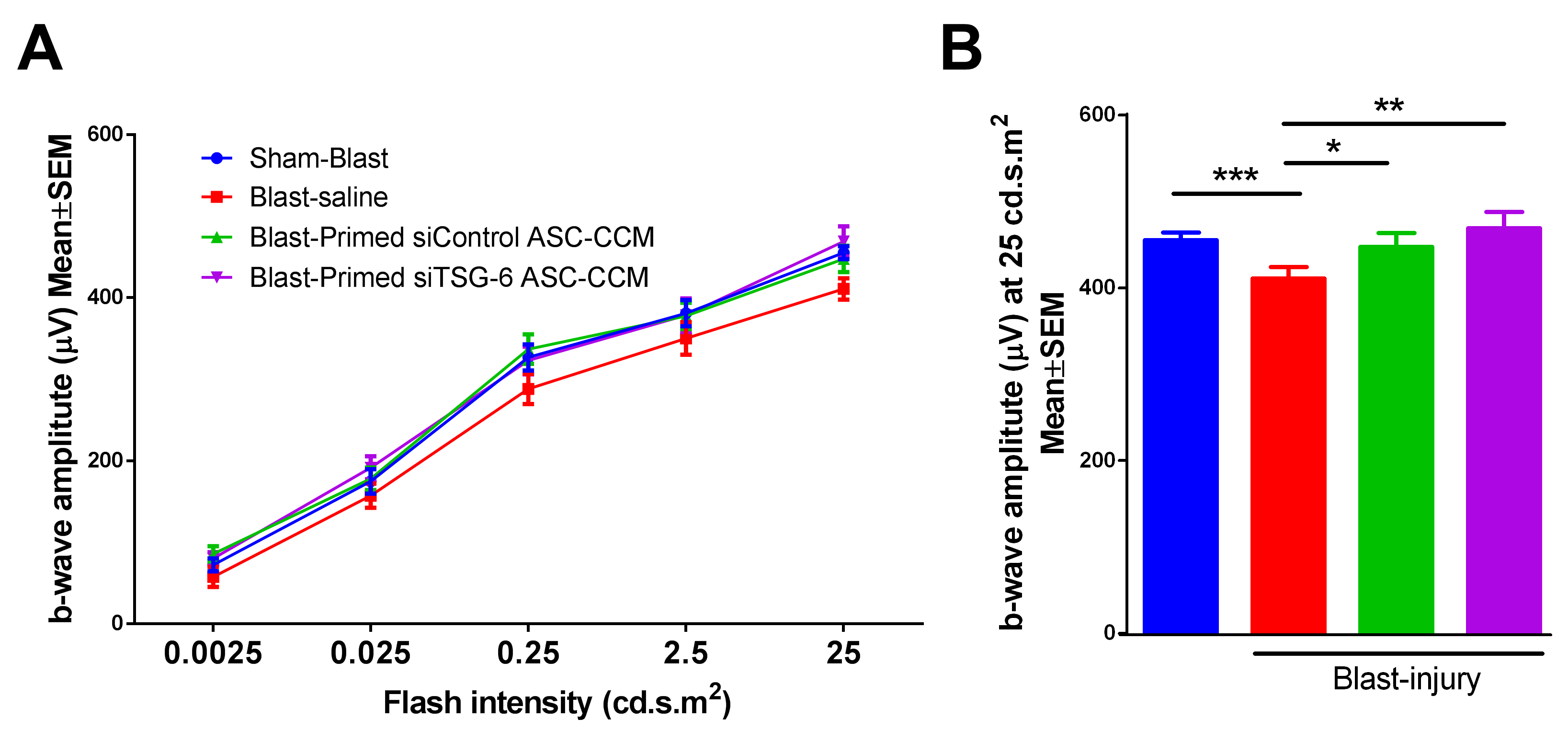

Supplement: Supplementary file 4 — Additional file 4: Figure S4. Depletion of TSG-6 from ASC-CCM improves retinal function and vision in blast injury mice. (A): b-wave amplitude measurement in mice at various flash intensities (B): b-wave amplitude at 25 cd.s.cm2 expressed as μV. Data represent combined Mean ± SEM from n = 8–19 animals/group of the left eye only performed in 2 separate batches. *p < 0.05; **p < 0.01; ***, p < 0.001. [file 13287_2019_1436_MOESM4_ESM.tif]
